# Supplementary material for: Determining the priority semen characteristics and appropriate age for genetic improvement in Thai native roosters
Source: PLoS One. 2025 Apr 2;20(4):e0319537. doi: 10.1371/journal.pone.0319537 (PMC11964250; doi:10.1371/journal.pone.0319537)
Supplement: Attachment — Submitted filename: Reponse to Reviewers.docx [file pone.0319537.s003.docx]

**Additional Editor Comments:**

Dear Dr., Wuttigrai Boonkum

Thank you for submitting your manuscript to PLOS ONE. After careful consideration, we have decided that your manuscript needs Major Revision.

**Comment editor**

The manuscript needs further interpretation of the results and in-depth discussion of the data.

Kind regards,

Prof. Lamiaa Mostafa Radwan, Ph.D.

Academic Editor

PLOS ONE

*Response:*	We are very grateful for the critical reading and your efforts to improve the quality of the manuscript. We hope that the manuscript in its revised form will please you. The responses to each comment are listed below.

**Reviewers’ comments:**

**Reviewer #1:** The paper evaluates genetic parameters for semen quality traits in local chickens. The methodology for variance components estimation is adequate but the design is poorly described (particularly populations structure and division into groups), the rationale for choosing traits and their economic weights in index is not correct. Recommendation of 10% selected is ad hoc without considering long term response to selection. The paper needs significant improvements in both analysis and writing.

**Response:**	Thank you for your insightful comments. We have addressed your concerns regarding the population structure, economic weights, and selection intensity as follows:

**Population structures and group division**: We have added a subheading, "Animal Samples and Management" (lines 122-137), to provide a detailed description of the animal grouping and sampling strategy " (lines 123-131).

**Economic Weights:** Direct economic valuation of semen traits proved impractical given data limitations. Therefore, a simplified relative weighting approach was employed, assigning weights to traits based on heritability, genetic correlations, and estimated economic importance. This method offers a practical alternative to complex bioeconomic modeling, enabling efficient genetic selection while optimizing for both direct and correlated genetic gains

**Selection Intensity (10%):** Our choice of a 10% selection intensity represents a balance between achieving rapid genetic improvement and mitigating the risks of inbreeding and genetic drift. This selection rate is commonly used in similar livestock improvement programs. To minimize inbreeding, we implemented a breeding strategy incorporating careful selection of breeding pairs, rigorous monitoring of inbreeding coefficients, and a plan to introduce new, unrelated genetic material into the population after 2-3 generations. This strategy is rewritten in detail in the Discussion section, where we outline our approach to maintaining genetic diversity over the long term; See lines 452-461 in the revised MS.

**Query 1:** Data access – there was no link or access to the data files provided

**Response 1:** The datasets used and/or analyzed during the current study are available from the corresponding author on reasonable request.

**Query 2:** L 40 Why does genetic correlation between the semen traits instead of genetic correlation to fertility determine which traits are important?

**Response 2:**	Direct selection for fertility is hampered by its low heritability and complex polygenic architecture. Focusing on the genetic correlations between semen traits provides a more efficient and practical alternative. This approach identifies groups of traits that influence the key physiological processes underlying fertility (e.g., spermatogenesis, sperm transport). Improving these readily measurable traits (motility, concentration, morphology) with stronger heritabilities is expected to positively impact overall fertility.

**Query 3:** L41-46 Not clear what the authors are trying to say

**Response 3:**	I have revised the sections you mentioned to enhance understanding by clarifying key concepts. See lines 42-47.

**Query 4:** L 71. Do you mean high breeding value for egg production? Roosters don’t have egg production

**Response 4:**	You raise a valid point. While roosters themselves do not lay eggs, they are crucial in determining the egg-laying potential of their female progeny. Line 71 refers to the indirect selection of superior egg-laying traits in female offspring through careful selection of roosters. This indirect selection is based on the rooster's breeding value for traits known to have a strong genetic correlation with egg production. We have rewritten the relevant section to clearly explain how the selection of roosters based on their breeding values for traits genetically correlated with egg production. The revised text also includes additional references to support this approach. See lines 65-73 in the revised MS.

**Query 5:** L73-76 Please clarify the thought process because these seem to be unrelated statements. Is the small number of offspring produced in order to constrain inbreeding rate or because of low fertility? Can pooled semen be used to increase fertility?

**Response 5:**	Thank you for pointing out the unclear connection between offspring numbers, inbreeding, and cost. The low number of offspring per hen is primarily due to the naturally low egg production rate of the native breed, not a deliberate inbreeding control strategy or AI-related fertility issues. This lower fecundity, as explained in lines 69-73, directly contributes to higher production costs.

**Query 6:** L79 Which quality trait or combination of traits has the strongest association with fertility?

**Response 6:**	We have expanded the dataset to include quality traits that have the strongest association with fertility. This is detailed in the revised manuscript, see lines 75-80.

**Query 7:** L87 “of age” not needed?

**Response 7:**	We have removed the word from "of age".

**Query 8:** L90 check language

**Response 8:**	We have revised the statement for clarity. See line 89-91.

**Query 9:** L127 The description of the design is not clear. What were the age groups if semen was collected at 32 weeks? If “the data were recorded for genetic analysis only when they were 32 weeks.” where does repeatability come from? Was it the same group of roosters recorded at different ages or is there a confounding between group and age? How many roosters were in each group? What was the genetic relationship between them? How deep was the pedigree?

**Response 9:**	For more clearly, we have added a subheading, "Animal Samples and Management" (lines 122-137), to provide a detailed description of the animal grouping and sampling strategy.

**Query 10:** L197 unfinished sentence

**Response 10:**	We have checked and correct the sentence. See line 196.

**Query 11:** L199-211 Not needed – these are textbook definitions of genetic parameters

**Response 11:**	As suggested, the textbook definitions of genetic parameters (lines 199-211) have been removed. The revised text (lines 197-198) now simply states that these parameters—heritability, repeatability, and genetic and phenotypic correlations—were estimated using a multi-trait animal model.

**Query 12:** The rationale for the index weights is incorrect. Economic weight should be based on how much more profit is generated by increasing that trait by one unit not by heritability and correlations between the indicator traits. If the goal trait is fertility subindex should be determined by optimizing w eights to maximize fertility.

**Response 12:**	We understand that determining economic values in livestock involves assigning monetary values to genetic traits based on their contribution to overall economic efficiency or profitability. However, with the current level of knowledge, accurately calculating the true monetary value of each semen trait may not be feasible. As a result, we opted for an alternative method that provides sufficient explanation of the results and can be effectively used for genetic selection of these traits. For this purpose, we selected the method of simple relative weight economic value.

Simple relative weight economic value is a simplified method for assigning economic values to traits in a breeding program. Rather than calculating precise monetary values, traits are assigned weights relative to one another based on their heritability, genetic correlations, and economic significance. These weights reflect the traits' contributions to overall profitability and efficiency in breeding programs. This approach is particularly useful when precise financial data or bioeconomic modeling is unavailable.

Additionally, this method does not require extensive data or complex modeling, making it practical for small-scale or resource-limited breeding programs. It enables breeders to make quick and effective selection decisions without complicated calculations.

In conclusion, heritability and genetic correlations play a vital role in calculating economic values and developing a reliable selection index. By considering both direct genetic improvements and correlated responses, this approach helps optimize genetic progress and achieve breeding goals efficiently.

**Query 13:** L 417-420 If young roosters have good fertility maybe selection focus should focus on improving persistency of fertility so older age records would be more relevant.

**Response 13:**	Thank you for raising this point. Our study indeed highlighted the importance of persistency in genetic traits; however, our findings demonstrated that the semen characteristics of semen volume, mass movement, and sperm concentration consistently showed the highest heritability across all rooster age groups. This consistency implies that these traits are genetically stable and do not decline significantly with age relative to others.

Moreover, our results suggest that selecting roosters at a younger age (Group 1: 32–52 weeks) is most advantageous. This is due to the higher genetic and phenotypic performance observed in this age group, coupled with the opportunity for rapid genetic improvement without the need to wait for older age records. These findings advocate for prioritizing young rooster traits, as they provide both accuracy and efficiency in genetic evaluation.

While this study focuses on the genetic improvement of semen traits in younger roosters, future research could investigate fertility persistency across a rooster's lifespan, incorporating GWAS and hormonal analyses to identify loci and physiological mechanisms affecting reproductive longevity. This would ultimately improve both early and long-term productivity. We have added more details in the revised manuscript's conclusion; see lines 467-474.

**Query 14:** L433 indicator traits not only have to have high heritability but also strong genetic correlation to the target trait

**Response 14:**	To enhance clarity and precision, we have revised this section accordingly. As a result, the sentence referencing this concept has been removed from the revised manuscript.

**Query 15:** L454 or make it more difficult if the direction is undesirable

**Response 15:**	As suggested, the revised text (lines 427-431) now explains that strong positive correlations between traits facilitate efficient selection, whereas strong negative correlations present challenges for simultaneous improvement.

**Query 16:** L484 high selection intensity results in higher inbreeding rate which can reduce long term response to selection, also other traits will be included in the selection index such as growth, meat quality, and feed efficiency. Expected results of alternative programs can be compared using deterministic or stochastic simulations

***Response 16:***	To minimize inbreeding, we implemented a breeding strategy incorporating careful selection of breeding pairs, rigorous monitoring of inbreeding coefficients, and a plan to introduce new, unrelated genetic material into the population after 2-3 generations. This strategy is rewritten in detail in the Discussion section, where we outline our approach to maintaining genetic diversity over the long term; See lines 452-461 in the revised MS.

**Reviewer #2:**

If the goal is to be published in a prestigious journal, it would be advisable to reconsider the analysis of the results, expand the scope of the research, perhaps by adding additional data or conducting broader comparisons with other types or breeds of poultry, and deepen the theoretical discussion.

***Response:*** We appreciate your insightful feedback and have made significant revisions throughout the manuscript to address your concerns. We divided the comments of reviewer into three sections: 1. Introduction; 2. Methods and results; and 3. Discussion and conclusion. Below, we address the points you raised related to the supporting factors for publication.

**Query 1:** **Introduction**

Points that may support publication in a prestigious journal:

The research objective is important and has practical value:

The study addresses a highly significant topic related to the genetic improvement of local poultry, which is of great interest in agriculture, genetics, and animal breeding fields.

The focus on improving semen quality in local poultry enhances productivity and profitability, providing clear practical value to the research.

***Response 1:***

The revised introduction transitions from being **descriptive** to being **impact-driven and application-focused**, addressing the reviewer’s concern by explicitly connecting the research findings to productivity, profitability, and practical benefits in the context of agriculture, genetics, and animal breeding. The revised introduction addresses the reviewer’s comment by emphasizing the **practical agricultural impact** and the **relevance of the research to genetics and animal breeding** in a more focused manner.

For agricultural impact, the revised version explicitly states that **reproductive efficiency and semen quality are pivotal to farm profitability** by ensuring continuous offspring generation, lowering production costs, and improving yields. This makes the agricultural significance more concrete and relatable for poultry producers.

For, relevance to Genetics and Animal Breeding, the revised version underscores how the research provides actionable insights into genetic improvement strategies, highlighting the development of a **selection index** for semen quality traits. It frames the research within the broader goals of **enhancing productivity through genetic selection**, a key aspect of animal breeding.

Meanwhile, the revised text positions semen quality as a **critical determinant of breeding efficiency**, linking traits like sperm motility and viability directly to fertility and productivity outcomes. This aligns with the reviewer's request to emphasize semen quality’s role in boosting agricultural efficiency.

**Query 2:** **Methods**

Advanced methodology:

The use of a multi-trait animal model and multivariate data analysis makes the methodology robust and meets the standards of advanced research.

Statistical analysis and the provision of estimates for heritability and repeatability support the research with precise data.

Practical and economic value:

The results help poultry breeders improve productivity and the quality of breeds in a scientific manner, which is important for journals with an applied focus.

Points that may hinder publication in a highly prestigious journal:

Strength of the results and depth of analysis:

Although the methodology is robust, the results may not be sufficient to support broader or deeper applications in the field. The heritability and repeatability results indicate low heritability in some traits (as mentioned, heritability values ranged between 0.043 and 0.161). These results may be perceived as having limited scientific and practical impact in the context of breed improvement.

The study does not appear to offer entirely new insights compared to previous studies, which could limit its appeal to high-impact journals. Prestigious journals typically seek research that presents innovative findings or new perspectives not previously known.

***Response 2:***

We appreciate the observation regarding the low heritability values reported in our study, as it raises an important consideration about the potential impact of these findings on breed improvement. While the heritability estimates for certain traits in our study ranged between 0.043 and 0.161, such values are not uncommon for fitness or reproductive traits, which are often influenced by environmental factors. Low heritability does not necessarily imply limited importance, as genetic progress can still be achieved with adequate selection intensity, large population sizes, or by leveraging advanced breeding strategies like genomic selection.

Traits with low heritability can still be targeted effectively in breeding programs, particularly when they have substantial economic or welfare significance. Additionally, these traits can be integrated into broader multi-trait selection indices to balance genetic gains across various objectives.

The robust methodology employed in this study ensures that the reported estimates are reliable and accurately reflect the genetic architecture of the traits under the studied conditions. This rigor provides a strong foundation for future studies and the development of tailored breeding strategies.

These findings also highlight the need for continued investigation, such as exploring genotype-by-environment interactions, incorporating larger datasets, or utilizing advanced genetic tools like whole-genome sequencing to identify causal variants. Such efforts could lead to improved understanding and more impactful applications in breed improvement.

In conclusion, the methodology remains robust with the use of a multi-trait animal model and multivariate data analysis, providing precise estimates for heritability and repeatability which supports our research findings. While the heritability estimates may be low, this study provides essential insights and a valuable starting point for developing strategies to enhance these traits. We believe the results contribute meaningfully to the growing body of knowledge in the field and offer avenues for practical application.

**Query 3:** Discussion and conclusion

Discussion and theoretical analysis:

The discussion section is relatively limited and does not delve deeply enough into the interpretation of results or link them effectively with previous studies. There is a need for a more in-depth analysis of the long-term effects of the results on poultry breeding and productivity improvement.

It may be necessary to strengthen the discussion of environmental factors and their impact on semen quality, and how these factors can be integrated with genetics to achieve better results.

Practical application:

Although the study focuses on improving semen quality, the specific practical applications are not clearly outlined. Prestigious journals prefer research that offers clear and practical solutions to existing challenges, which may require further development in this manuscript.

Conclusion:

In light of the above points, the manuscript makes a good scientific contribution but may not be sufficient for publication in a highly prestigious journal due to the relative weakness of the results and limited analysis. The findings do not present groundbreaking advancements or sufficiently novel discoveries to attract the attention of high-impact journals. However, the research may be suitable for publication in a good journal of medium level, or one with an applied interest in animal breeding or poultry genetics.

If the goal is to publish in a prestigious journal, it would be advisable to reconsider the analysis of the results, expand the scope of the research, perhaps by adding additional data or conducting broader comparisons with other types or breeds of poultry, and deepen the theoretical discussion.

***Response 3:***

By addressing each of the reviewer's comments with specific revisions and enhancements, we have improved the manuscript's depth and clarity, we have revised the discussion session to a more comprehensive understanding of semen quality and its implications for poultry breeding, thereby making the case for the manuscript's suitability for publication in a prestigious journal.

**1. Depth of Discussion and Interpretation of Results:**

- **Revised Content:**
  - The discussion section has been significantly expanded to include a more comprehensive interpretation of findings.
  - Additional literature references have been incorporated to establish clear comparisons between our results and previous studies.
  - Specific examples from studies comparing different rooster breeds were included to enhance the understanding of how our findings align with or deviate from existing knowledge.

**2. Long-term Effects on Poultry Breeding and Productivity:**

- **Revised Content:**
  - We have added a thorough analysis of the long-term implications of semen quality on breeding outcomes and overall productivity.
  - This includes strategies for optimizing rooster selection for sustained reproductive efficiency.

**3. Environmental Factors and Their Integration with Genetics:**

- **Revised Content:**
  - We strengthened the discussion around how environmental factors, such as nutrition and housing, influence semen quality.
  - We emphasized the necessity of optimizing these conditions alongside genetic factors to maximize breeding outcomes.

**4. Practical Applications:**

- **Revised Content:**
  - Specific recommendations for poultry breeders have been added to the discussion, presenting actionable strategies that can be applied to improve semen quality traits.
  - We also discussed the economic benefits of prioritizing young roosters based on semen quality criteria.

**5. Overall Contribution and Publication Suitability:**

- **Revised Content:**
  - Adjustments were made throughout the manuscript to enhance clarity, rigor, and the overall contribution of the research.
  - The discussion on the significance of findings was amplified to more clearly articulate their potential impact on the field of poultry genetics and breeding.

**6. Future Research Directions:**

- **Revised Content:**
  - A segment was added that outlines potential future research avenues, emphasizing the need for studies that further investigate the genetic and environmental factors influencing semen quality.
  - Suggestions for integrating genome-wide association studies (GWAS) were included to encourage deeper genetic analysis in future research.
