# Supplementary material for: Determining the priority semen characteristics and appropriate age for genetic improvement in Thai native roosters
Source: PLoS One. 2025 Apr 2;20(4):e0319537. doi: 10.1371/journal.pone.0319537 (PMC11964250; doi:10.1371/journal.pone.0319537)
Supplement: Attachment — Submitted filename: Reponse_to_Reviewers_auresp_2.docx [file pone.0319537.s004.docx]

**Additional Editor Comments:**

Dear Dr., Wuttigrai Boonkum

Thank you for submitting your manuscript to PLOS ONE. After careful consideration, we have decided that your manuscript needs Minor Revision.

**Comment editor**

It is necessary to show the scientific and economic importance of the manuscript in abstract and discussion of results

Kind regards,

Prof. Lamiaa Mostafa Radwan, Ph.D.

Academic Editor

PLOS ONE

**Response:** We are very grateful for the critical reading and your efforts to improve the quality of the manuscript. We have revised the abstract to highlight its scientific and economic significance, as shown below and in lines 20-54. This revised abstract provides crucial insights into the genetic evaluation of semen traits in Thai native grandparent roosters, offering a foundation for targeted genetic improvement. By enhancing reproductive efficiency through selective breeding, the study not only accelerates genetic progress but also boosts the economic sustainability of native chicken production, benefiting smallholder farmers and supporting the poultry industry's long-term viability in tropical regions.

“Semen characteristics are crucial indicators of reproductive success and directly influence the production efficiency of native chickens, which play a vital role in sustainable poultry production systems in Thailand. Key semen traits, including mass movement, semen pH, semen volume, sperm viability, sperm abnormalities, and sperm concentration, are routinely evaluated to assess the reproductive performance of Thai native grandparent roosters. Improved reproductive efficiency in these chickens can lead to increased fertility rates, better hatchability, and enhanced productivity, significantly benefiting smallholder farmers and the poultry industry. To enhance the accuracy and efficiency of evaluating the genetic potential of grandparent roosters, this study aimed to compare rooster age groups, estimate genetic parameters and breeding values, and develop an optimal selection index for semen traits. A total of 3,475 test-day records of six semen traits from Thai native grandparent roosters (Pradu Hang Dum), obtained from the Khon Kaen University native chicken experimental farm, were analyzed using a multi-trait animal model with average information restricted maximum likelihood (AI-REML). The heritability estimates for semen volume, mass movement, sperm concentration, sperm abnormalities, sperm viability, and semen pH across age groups were low, ranging from 0.128 to 0.161, 0.102 to 0.115, 0.101 to 0.111, 0.090 to 0.091, 0.067 to 0.083, and 0.043 to 0.057, respectively. Repeatability estimates ranged from low to moderate, between 0.119 and 0.384 for all traits and age groups. Genetic correlations among semen traits ranged from -0.332 to 0.580, -0.554 to 0.640, and -0.631 to 0.773 for rooster age Groups 1, 2, and 3, respectively. Based on heritability and genetic correlations, the three most important semen traits identified were semen volume, mass movement, and sperm concentration. The study revealed a strong relationship between rooster age and semen quality, with the highest selection index values observed in the youngest age group (32–52 weeks). This finding highlights the potential for genetic improvement by implementing a selective breeding program targeting the top 10% of young roosters based on the developed selection index. Such a strategy would significantly accelerate genetic progress in semen traits, improving reproductive efficiency and boosting the economic viability of native chicken production. Furthermore, these findings contribute to a deeper understanding of genetic evaluation in native chicken populations and can serve as a model for developing sustainable breeding programs for other poultry breeds under tropical conditions.”

In addition, we have also revised the discussion section to include original ideas relevant to the scientific community, producers, and breeders, while highlighting the economic significance of this study. See the detail below and lines 506-544 in the revised manuscript.

“This study emphasizes the importance of considering age as a critical factor in genetic evaluation models for reproductive traits. The significant decline in semen quality with increasing age underscores the need for further research into the physiological mechanisms behind age-related changes in male fertility. Additionally, these findings open opportunities for exploring strategies to extend the productive lifespan of roosters through dietary, hormonal, or environmental interventions. The study also demonstrates the utility of genetic parameters and selection indices tailored to specific age groups. By focusing on younger roosters with optimal semen quality, future research could refine breeding objectives to maximize genetic gains in reproductive traits, ensuring the long-term sustainability of native chicken populations.

From a practical perspective, the results offer valuable insights for poultry breeders and producers. Younger roosters demonstrate superior reproductive efficiency and provide a cost-effective way to enhance flock fertility and productivity. Adopting age-specific breeding strategies, such as prioritizing roosters aged 32–52 weeks for semen collection and artificial insemination, can significantly improve fertility rates and hatchability in commercial operations. Furthermore, the study highlights the economic advantages of optimizing reproductive performance in Thai native chickens. By selectively breeding roosters with superior semen traits, producers can achieve greater genetic progress, resulting in improved flock productivity and profitability. This approach aligns with sustainable poultry production systems, particularly in smallholder farming contexts where native chickens play an essential role in household income and food security.

The economic importance of this study goes beyond the immediate advantages for smallholder farmers. Improving reproductive efficiency helps reduce input costs by minimizing the need to maintain older, less productive roosters, thereby enhancing overall production efficiency. Moreover, since Thai native chickens play a vital role in sustainable poultry production systems in tropical regions, enhancing their genetic potential supports the preservation of local breeds and biodiversity. These findings also provide a model for improving other tropical poultry breeds, demonstrating how scientific advancements can be applied to develop practical solutions that address both economic and environmental challenges (64–66). In summary, this study establishes a framework for age-specific genetic improvement programs that benefit not only the poultry industry but also the scientific community. By combining genetic insights with practical breeding strategies, it offers a pathway to achieving sustainable and economically viable native chicken production systems in tropical environments.”

**Reviewers’ comments:**

**Reviewer 2**

Accept

**Response:** Thank you very much for your support.

**Reviewer 3**

2- The present article delineates a scientific study that yielded predictable and consistent results with existing literature.

**Response:** We are pleased that the consistency and predictability of our results were recognized. This further supports the reliability and relevance of our research methodology.

3- I am does not possess sufficient information regarding the appropriateness of statistical analysis.

**Response:** We would like to emphasize that this study highlights the importance of carefully selecting suitable tools for genetic estimation to ensure accurate, reliable, and meaningful results for future research.

4-The authors have made fully available all the data underlying the findings in their article.

**Response:** Thank you for acknowledging that all data underlying the findings in our article have been made fully available. Ensuring data transparency and accessibility is an important aspect of our work, and we are pleased this has been recognized.

5-The manuscript is presented in a lucid manner and is written in standard English.

**Response:** Thank you for your positive feedback on the clarity and language of our manuscript. We are glad to hear that the manuscript is presented lucidly and meets the standard for English writing. We appreciate your acknowledgment, as we have strived to ensure the content is accessible and well-written for the readership.

The objective of this study was to develop a suitable selection index for semen characteristics by age in native Thai grandparent roosters. The findings from this research, although predictable, may provide a framework for the genetic improvement of domestic chickens.

**Response:** Thank you very much for your support.

**Reviewer 4**

After carefully revised the Manuscript Number PONE-D-24-30630R1 under the title of "Determining the priority semen characteristics and appropriate age for genetic improvement in Thai native roosters"

I think the manuscript lacks originality and creativity. The authors must introduce new and original ideas to the scientific community, producers, and breeders. However, the manuscript just introduces and offers traditional information and results, which are known to all scientists and breeders.

**Response:** Thank you for your insightful comments. We have also revised the discussion section to include original ideas relevant to the scientific community, producers, and breeders, while highlighting the economic significance of this study. See the detail below and lines 506-544 in the revised manuscript.

“This study emphasizes the importance of considering age as a critical factor in genetic evaluation models for reproductive traits. The significant decline in semen quality with increasing age underscores the need for further research into the physiological mechanisms behind age-related changes in male fertility. Additionally, these findings open opportunities for exploring strategies to extend the productive lifespan of roosters through dietary, hormonal, or environmental interventions. The study also demonstrates the utility of genetic parameters and selection indices tailored to specific age groups. By focusing on younger roosters with optimal semen quality, future research could refine breeding objectives to maximize genetic gains in reproductive traits, ensuring the long-term sustainability of native chicken populations.

From a practical perspective, the results offer valuable insights for poultry breeders and producers. Younger roosters demonstrate superior reproductive efficiency and provide a cost-effective way to enhance flock fertility and productivity. Adopting age-specific breeding strategies, such as prioritizing roosters aged 32–52 weeks for semen collection and artificial insemination, can significantly improve fertility rates and hatchability in commercial operations. Furthermore, the study highlights the economic advantages of optimizing reproductive performance in Thai native chickens. By selectively breeding roosters with superior semen traits, producers can achieve greater genetic progress, resulting in improved flock productivity and profitability. This approach aligns with sustainable poultry production systems, particularly in smallholder farming contexts where native chickens play an essential role in household income and food security.

The economic importance of this study goes beyond the immediate advantages for smallholder farmers. Improving reproductive efficiency helps reduce input costs by minimizing the need to maintain older, less productive roosters, thereby enhancing overall production efficiency. Moreover, since Thai native chickens play a vital role in sustainable poultry production systems in tropical regions, enhancing their genetic potential supports the preservation of local breeds and biodiversity. These findings also provide a model for improving other tropical poultry breeds, demonstrating how scientific advancements can be applied to develop practical solutions that address both economic and environmental challenges (64–66). In summary, this study establishes a framework for age-specific genetic improvement programs that benefit not only the poultry industry but also the scientific community. By combining genetic insights with practical breeding strategies, it offers a pathway to achieving sustainable and economically viable native chicken production systems in tropical environments.”
