# Supplementary material for: Determining the priority semen characteristics and appropriate age for genetic improvement in Thai native roosters
Source: PLoS One. 2025 Apr 2;20(4):e0319537. doi: 10.1371/journal.pone.0319537 (PMC11964250; doi:10.1371/journal.pone.0319537)
Supplement: Attachment — Submitted filename: Report manuscript reviewer.docx [file pone.0319537.s001.docx]

**Based on a thorough review of the manuscript, several factors influence the decision of whether it is suitable for publication in a prestigious journal. Here is a precise scientific analysis of whether it deserves publication in a high-ranking journal:**

Points that may support publication in a prestigious journal:

The research objective is important and has practical value:

The study addresses a highly significant topic related to the genetic improvement of local poultry, which is of great interest in agriculture, genetics, and animal breeding fields.

The focus on improving semen quality in local poultry enhances productivity and profitability, providing clear practical value to the research.

Advanced methodology:

The use of a multi-trait animal model and multivariate data analysis makes the methodology robust and meets the standards of advanced research.

Statistical analysis and the provision of estimates for heritability and repeatability support the research with precise data.

Practical and economic value:

The results help poultry breeders improve productivity and the quality of breeds in a scientific manner, which is important for journals with an applied focus.

Points that may hinder publication in a highly prestigious journal:

Strength of the results and depth of analysis:

Although the methodology is robust, the results may not be sufficient to support broader or deeper applications in the field. The heritability and repeatability results indicate low heritability in some traits (as mentioned, heritability values ranged between 0.043 and 0.161). These results may be perceived as having limited scientific and practical impact in the context of breed improvement.

The study does not appear to offer entirely new insights compared to previous studies, which could limit its appeal to high-impact journals. Prestigious journals typically seek research that presents innovative findings or new perspectives not previously known.

Discussion and theoretical analysis:

The discussion section is relatively limited and does not delve deeply enough into the interpretation of results or link them effectively with previous studies. There is a need for a more in-depth analysis of the long-term effects of the results on poultry breeding and productivity improvement.

It may be necessary to strengthen the discussion of environmental factors and their impact on semen quality, and how these factors can be integrated with genetics to achieve better results.

Practical application:

Although the study focuses on improving semen quality, the specific practical applications are not clearly outlined. Prestigious journals prefer research that offers clear and practical solutions to existing challenges, which may require further development in this manuscript.

Conclusion:

In light of the above points, the manuscript makes a good scientific contribution but may not be sufficient for publication in a highly prestigious journal due to the relative weakness of the results and limited analysis. The findings do not present groundbreaking advancements or sufficiently novel discoveries to attract the attention of high-impact journals. However, the research may be suitable for publication in a good journal of medium level, or one with an applied interest in animal breeding or poultry genetics.

If the goal is to publish in a prestigious journal, it would be advisable to reconsider the analysis of the results, expand the scope of the research, perhaps by adding additional data or conducting broader comparisons with other types or breeds of poultry, and deepen the theoretical discussion.
